# Polyphenols and Visual Health: Potential Effects on Degenerative Retinal Diseases

**DOI:** 10.3390/molecules26113407

**Published:** 2021-06-04

**Authors:** Pol Fernandez-Gonzalez, Aina Mas-Sanchez, Pere Garriga

**Affiliations:** Grup de Biotecnologia Molecular i Industrial, Centre de Biotecnologia Molecular, Departament d’Enginyeria Química, Universitat Politècnica de Catalunya, Edifici Gaia, 08222 Terrassa, Spain; pol.fernandez.gonzalez@upc.edu (P.F.-G.); aina.mas.sanchez@upc.edu (A.M.-S.)

**Keywords:** flavonoids, retinal degenerative diseases, retinitis pigmentosa, protein folding, ligand binding, rhodopsin

## Abstract

Dietary polyphenols are a group of natural compounds that have been proposed to have beneficial effects on human health. They were first known for their antioxidant properties, but several studies over the years have shown that these compounds can exert protective effects against chronic diseases. Nonetheless, the mechanisms underlying these potential benefits are still uncertain and contradictory effects have been reported. In this review, we analyze the potential effects of polyphenol compounds on some visual diseases, with a special focus on retinal degenerative diseases. Current effective therapies for the treatment of such retinal diseases are lacking and new strategies need to be developed. For this reason, there is currently a renewed interest in finding novel ligands (or known ligands with previously unexpected features) that could bind to retinal photoreceptors and modulate their molecular properties. Some polyphenols, especially flavonoids (e.g., quercetin and tannic acid), could attenuate light-induced receptor damage and promote visual health benefits. Recent evidence suggests that certain flavonoids could help stabilize the correctly folded conformation of the visual photoreceptor protein rhodopsin and offset the deleterious effect of retinitis pigmentosa mutations. In this regard, certain polyphenols, like the flavonoids mentioned before, have been shown to improve the stability, expression, regeneration and folding of rhodopsin mutants in experimental in vitro studies. Moreover, these compounds appear to improve the integration of the receptor into the cell membrane while acting against oxidative stress at the same time. We anticipate that polyphenol compounds can be used to target visual photoreceptor proteins, such as rhodopsin, in a way that has only been recently proposed and that these can be used in novel approaches for the treatment of retinal degenerative diseases like retinitis pigmentosa; however, studies in this field are limited and further research is needed in order to properly characterize the effects of these compounds on retinal degenerative diseases through the proposed mechanisms.

## 1. Introduction

Different studies have reported that dietary polyphenols exert protective and beneficial effects against chronic diseases such as neurodegenerative and cardiovascular diseases, cancer, and diabetes [1]; however, the mechanisms underlying these benefits are far from being completely understood and more research is needed in order to define them. Despite all the benefits polyphenols can provide, there are some important aspects that have to be taken into account when discussing their physiological effects. These compounds have low oral bioavailability and other properties like their physicochemical stability, gastrointestinal absorption, and metabolism are important to ensure an effective action [2,3].

Despite the existent gap of knowledge in their action mechanism, the World Health Organization has recommended to increase the intake of fruit, vegetables, and fiber due to the high number of plant-derived components [4] with polyphenols having an important role, since they may confer health benefits related to non-communicable diseases (NCDs) [5,6]. Although associating polyphenols with specific diseases is challenging [7], some promising results have been obtained in different observational studies regarding polyphenols and certain NCDs [8,9], including some visual diseases. For this reason, the implication of polyphenols in health and disease states needs to be studied and better defined because of the expected positive impact on human health.

The main purpose of this review is to provide a synthetic account of some of the potential health benefits that polyphenols can have to improve human quality of life and to ameliorate the progression of some diseases, with a special emphasis on visual disorders. The use of a number of ubiquitous polyphenolic compounds is investigated here as potential treatments for a wide range of pathological conditions. Herein, we focus on the effects of these compounds on retinal functioning and particularly on their potential use to counteract retinal protein mutations, like those associated with retinal degenerative diseases like retinitis pigmentosa (RP). There is a clear need for more clinical trials to unravel their physiological significance as only a small number of studies have been conducted on the effects of polyphenols in human vision and their results need further validation.

## 2. Methods

This article is a literature review of the effect polyphenols can exert on some visual diseases, especially their impact on retinal degenerative diseases. Given the new properties that have been given to polyphenols over the years, the main purpose of our research is to show that these compounds may have positive effects on human visual health and also encourage researchers to inquire into this field. The different studies in this review (randomized control trials, meta-analysis, reviews, and experimental and observational studies) were identified by searching the PubMed and Cochrane databases up to and including February 2021. The following medical subject heading keywords were used: “polyphenols”, “flavonoids”, “retinal degenerative diseases”, and “retinitis pigmentosa”. Relevant papers were identified and selected independently by two authors following the inclusion criteria, i.e., original full-text articles that were written in English (including clinical and preclinical studies). On the other hand, the exclusion criteria included papers and articles written in other languages than English.

## 3. Polyphenols as Repurposed Drugs

**Figure 1:**
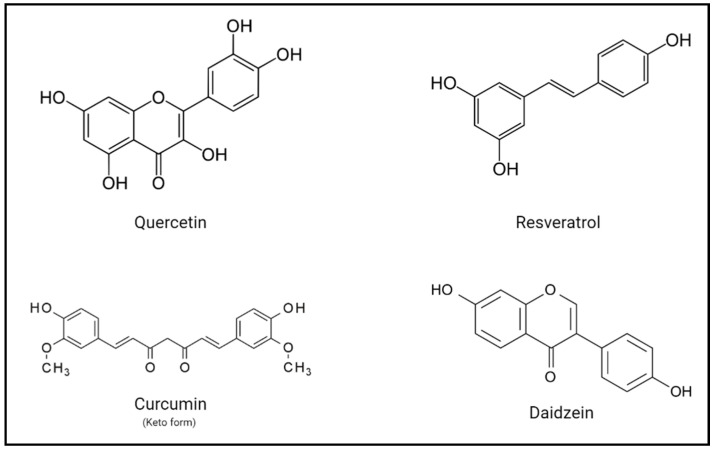
Structures of representative and abundant polyphenols from different subfamilies. Quercetin is a typical flavonoid found in many products. Resveratrol is a natural polyphenolic phytoalexin. Curcumin is derived from the rhizome of turmeric and is usually found in its keto form. Finally, daidzein is one of the most common isoflavones.

Polyphenols or dietary phenolic compounds are known as the largest group of phytochemicals [10] and are a group of natural compounds sharing common structural features (Figure 1). Currently, there is a renewed interest in this wide family of natural compounds due to the potential roles regarding human health and disease states. Different lines of evidence, derived from sustained work in the last several years, provide support for an important role for polyphenols both in helping maintain a healthy life-style and in the prevention of prevalent diseases like cancer, cardiovascular and neurodegenerative diseases [11,12,13]. Specifically, several studies have suggested that the consumption of different polyphenols from natural sources such as fruit and vegetables can contribute to preserving vision and can even reverse visual impairment in certain visual disorders [14,15].

The polyphenol superfamily includes a large number of sub-families, among which we can find flavonoids, phenolic acids, stilbenes, and lignans [16]. In fact, they constitute a group of natural products in the plant kingdom that is one of the most numerous and ubiquitously distributed. One of the most studied groups, from these different sub-classes, is that of flavonoids, comprising over 4000 members [17]. Flavonoids have a characteristic structure of a 15-carbon skeleton of a chromane ring attached to another aromatic ring [18]. The biosynthesis of these complex polyphenols is linked to primary metabolism [10]. Flavonoids are stored, in their native state, in plants as glycoside and non-glycosylated conjugates and can be absorbed by the small intestine and readily metabolized, once ingested, by phase II enzymes. After this biochemical process, the resulting moieties can enter systemic circulation [19,20].

It should be noted that not all flavonoids are absorbed by the small intestine. A large number of them enter the large intestine, where the deconjugated metabolites are degraded by the colonic microbiota into molecules like phenolic acids that can be easily absorbed [19].

One of the main proposed biological actions of polyphenols is associated with their antioxidant power within living cells; however, detailed investigations indicate that these effects, in many tissues, may not be as relevant as previously suggested. This is due to the fact that in many tissues it is difficult for these compounds to reach the threshold concentration needed to exert any significant biological effect [21,22]. Nonetheless, recent studies have suggested that polyphenols may have significant effects on human health, such as anti-inflammatory, anti-microbial, and tumor-suppressing properties [23,24,25].

The diversity of polyphenolic compounds of natural origin, their chemical lability, and their complex bioavailability patterns consequently necessitates stringent evaluation of the physiological effects of these compounds, and such evaluations are not always available. These evaluations are absolutely needed for later use in therapeutic applications.

## 4. Implications and Potential Benefits of Polyphenols on Human Health

As already discussed, polyphenols have been well characterized for their antioxidant effects, but their physiological relevance has been questioned due to the limited bioavailability that renders relatively low concentrations which may hamper achieving significant in vivo effects [21,22]; however, different alternative molecular mechanisms in which polyphenols appear to have a role have been identified and this gives these compounds another set of properties that may represent benefits for human health. These include different actions both at the intra- and inter-cellular signaling pathways levels, like, for example, regulating nuclear transcription factors and fat metabolism and modulating the synthesis of inflammatory mediators like cytokines, tumor necrosis factor α, interleukin-1β, and interleukin-6 [26,27]. As a general overview, different studied flavonoids have been shown to play different roles in cellular processes, such as increasing insulin secretion, reducing apoptosis, promoting β-cell proliferation, and reducing inflammation and oxidative stress in some cells [28]. All these effects play a role in different processes, such as glucoregulation, and show that flavonoids can have favorable effects on diabetes and obesity prevention and control [29,30,31].

Despite all the potential beneficial effects of polyphenols proposed to date, a key aspect to consider concerns the effective concentration in the human body of these compounds and the amount of natural food that needs to be consumed to reach such a concentration. In many cases, the required polyphenol quantity needed to exert a certain physiological function can be attained by the consumption of common foods in appropriate amounts in a normal diet, and, in such cases, toxicity is avoided. In other cases, where the potential beneficial effects can be foreseen, the consumption of polyphenol-containing foods has to be increased and in principle no adverse effects are expected if this increase is carefully planned. Finally, if the physiologically active concentration cannot be reached with common food ingestion, then dietary supplementation or pharmacological approaches may be necessary. This can lead to an increase in adverse secondary effects that would require a strict follow-up of the intake and a proper control of the dose regime [30].

Polyphenols are mainly provided by the intake of different food sources, such as coffee, tea, cocoa, and apples and they have been associated with several potential health benefits [5,6,11,14,32,33,34,35,36]. In fact, polyphenols have been mechanistically involved in glucose metabolism, platelet function, endothelial function, blood pressure, inflammation, and cholesterol levels, among others [37,38]. This variety of cellular functions that may be affected by the action of polyphenols provides an excellent platform for the development of effective health prevention strategies as well as novel therapeutic approaches not only for prevalent NCDs but even for genetic hereditary rare pathological conditions [5,6].

Some of the evidence regarding the beneficial effect of polyphenols on human health comes from observational studies and this implies taking several factors into consideration when extracting meaningful conclusions when interpreting experimental data. For example, a high intake of polyphenols from different foods may be balanced by a reduced intake of otherwise potentially harmful foods of an animal origin; however, observational studies can be helpful in many cases, e.g., in the formulation of hypotheses that will then be included into controlled intervention studies. In fact, observational studies regarding this matter should be complemented and supported by rigorous and wide clinical studies that evaluate the hypothesis that dietary phenolics have a positive role in improving human health and preventing disease states [28].

In addition to all the effects on cardiometabolic health, polyphenols are also thought to have a beneficial role on cognitive function. For example, some longitudinal studies show that regular dietary chocolate consumption can reduce the risk of cognitive decline [39,40]. Studies of other food sources like tea show that its consumption can help lowering the risk of cognitive impairment, reduce the risk of depression, and have protective effects against some diseases like Parkinson’s disease [41,42,43,44].

From all polyphenol types, dietary flavonoids can also have beneficial effects in retinal degenerative diseases like retinitis pigmentosa, where mutations in retinal proteins can cause photoreceptor cell death and vision loss, eventually leading to blindness. In fact, the flavonoid quercetin was found to have an effect on the conformational stability and function of the visual G protein-coupled receptor (GPCR) rhodopsin (Rho) [45]. These results suggest that quercetin can have a positive effect on the stability and conformational properties of the G90V Rho RP mutant. These results emphasize that other roles, in addition to their established antioxidant effect, can be envisaged for flavonoids and other polyphenols. The effect on retinal Rho suggests an effect at the receptor level that deserves further investigation. These results open a new frame of possibilities to use this and other flavonoids, possibly in combination with specific retinoids, in order to treat retinal degeneration associated with RP. This strategy could also be used to overcome the mutational effect associated with different pathological conditions in other members of the GPCR superfamily [45].

Polyphenolic compounds, and particularly flavonoids, are good prospects for treating or ameliorating the progression of human diseases, in addition to their established antioxidant potential that is considered important as part of a healthy lifestyle.

## 5. Vertebrate Rho and Retinal Degeneration

Photoreceptor cells are primary sensory neurons in the retina that detect light and convert this energy into nerve impulses that lead to visual perception in the brain. Light absorption occurs in the two types of photoreceptor cells present in the retina, rods, and cones, and this process is mediated by the visual pigments contained in them. The main photoreceptor protein present in the retina is Rho [46,47]. Both Rho and cone opsins belong to the family of GPCRs and are composed of an apoprotein, opsin, and a chromophore, namely, 11-*cis*-retinal (11CR) [48,49,50].

### 5.1. Rho as a GPCR

GPCRs are membrane proteins consisting of a single polypeptide chain structured in a helical architecture. All members of the GPCRs superfamily share a common structure with seven hydrophobic helical segments connected by three extracellular and three intracellular loops. A fourth loop is formed by joining the C-terminal segment and the lipid bilayer through cysteine palmitoylation [51]. The *N*-terminal part and extracellular loops recognize a wide variety of ligands and modulate their binding to the receptor. The seven transmembrane segments form the structural nucleus and transduce extracellular signals into the internal domain by conformational changes. The intracellular part interacts with cytosolic G proteins, arrestins, GPCR kinases, and other signaling effectors [52,53,54].

GPCRs respond to a large number of endogenous allosteric modulators. These modulators regulate receptor function by binding to alternative regions of the conventional orthosteric site. While also permitting the binding of orthosteric ligands, they can modulate the affinity and efficacy of the orthosteric ligand [55]. Orthosteric and allosteric ligands that act on the same GPCR can participate in different regulatory and signaling pathways by interacting with effectors and regulatory proteins. Both can select different parts of these signaling and regulation pathways by establishing different receptor conformations, a phenomenon called functional selectivity. The pathways where the ligand–receptor complex is involved will determine the physiological effects of the ligand [56,57,58].

There is a growing interest in ligands that bind to allosteric sites, as they may be potentially more selective than orthosteric ligands. This is because their binding occurs in less conserved regions, making them promising therapeutic substances with a lower risk of overdose and fewer adverse effects [51].

Rho is the prototypical GPCR of the human retina that mediates dim light vision. Rho absorbs a quantum of light and converts it into an electrical signal that is transmitted to the brain by means of the visual phototransduction process. This process involves a specific G protein, transducin, and Rho kinase and arrestin, among other proteins that are key players in the correct functioning of the visual system [59].

### 5.2. Visual Phototransduction

Rho converts photons into chemical signals that can trigger biological processes by allowing the brain to perceive light stimuli [59]. Rho is bound to 11CR in its dark-adapted (ground-state inactive) conformation. 11CR is a derivative of vitamin A that has a very fast response and a high quantum yield upon light absorption in its isomerization reaction to all-*trans*-retinal (ATR). These photochemical properties, which are unique to the chemical structure of 11CR, have preserved this small molecule to remain invariant through evolution of vision and opsin moieties have adapted their amino acid sequences to different environments by evolutionary selection. The 11CR chromophore is covalently attached via a protonated bond from the Schiff base to K296 at the seventh transmembrane helix of Rho [60]. The transduction of signals in the visual system comprises two processes: (i) the activation of Rho by a photon of light that leads to a conformational change (involving helical rearrangements of the protein) and (ii) a deactivation step, or signal shut-off, that involves Rho kinase and arrestin to eventually regenerate Rho to its original inactive dark state [61,62].

**Figure 2:**
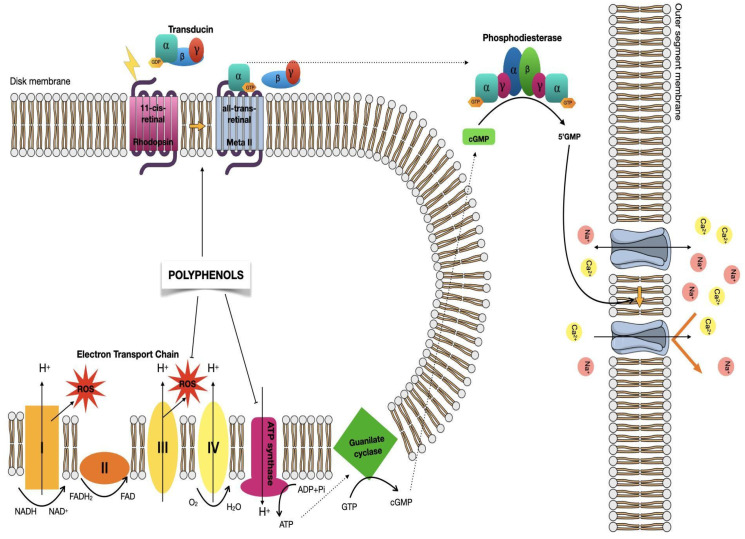
Visual phototransduction. Rho activates light causing isomerization of 11CR to ATR. The active form of Rho (meta II) interacts with Gt (composed of the α, β, and γ subunits) activating it and causing the exchange of GDP for GTP and the dissociation of the α subunit. This, in turn, activates cyclic guanosine monophosphate phosphodiesterase (cGMP), promoting the hydrolysis of cGMP and its conversion to 5′-GMP. Reduction in cytoplasmic cGMP concentration leads to closure of transmembrane channels by blocking the internal flow of Na^+^ and Ca^2+^ and leading to the hyperpolarization of the cell [62,64,65]. The electron transport chain and the ATP synthase present in the rod photoreceptors disks produce energy needed for the process, this energy production results in the production of reactive oxygen species (ROS) which can lead to cell damage. [67]. Polyphenols can act in different ways at the cellular level, they seem to be able to stabilize mutated Rho, can inhibit the ATP synthase or help prevent the ROS damage with their antioxidative effect.

In vertebrates, the visual signal begins with the absorption of photons by 11CR that cause the isomerization of the 11-12 double bond to yield the ATR stereochemical configuration [49,63]. Complete chromophore isomerization causes a change in the conformation of the protein, making the coupling to opsin less energetically favorable and promoting ATR release from the retinal binding pocket. This active photoilluminated conformation, termed metarhodopsin II (meta II), activates the signal transduction process by binding to the heterotrimeric G-protein transducin (Gt) and activating it by promoting the dissociation of α from the βγ subunits. This, in turn, activates a cyclic guanosine monophosphate (cGMP) phosphodiesterase that hydrolyzes cGMP causing the closure of the ion channels of the membrane and the subsequent hyperpolarization of the cell (Figure 2) [62,64,65]. The potential difference in photoreceptor cells is transferred through the synaptic terminal to second-order neurons of the retina [66].

After the activation of Rho, a constant supply of 11CR is required. This is obtained from the retinoid cycle, which is an enzymatic pathway occurring at the photoreceptors and the retinal pigment epithelium (RPE). The process re-isomerizes the entire ATR back to 11CR so that this newly produced 11CR can recombine with opsin to regenerate Rho [68].

Both the function and integrity of photoreceptors are crucial to vision. Mutations that affect the function of these receptors, or other factors that can alter the phototransduction process, can cause visual dysfunction or a loss of vision. Defects in other types of retinal cells (such as RPE) can also cause visual cycle dysfunction [66,69,70]. There is a high concentration of Rho in the retina, so intense light can cause a local concentration of free ATR that is toxic to cells, which can cause severe retinal degeneration and even eventually lead to complete blindness [46,66,71]. In addition, when 11CR is not effectively recombined with opsin, high concentrations of non-regenerated opsin can promote and enhance retinal degeneration processes [72,73,74,75,76].

The death of photoreceptors caused by persistent exposure to high light intensities is associated with changes in cellular metabolism and the overproduction of reactive oxygen species (ROS) that can cause cell damage [77,78]. As a result, apoptotic pathways are activated, resulting in the death of photoreceptor cells [78]. Furthermore, the toxic effect produced by light results in the expression of pro-inflammatory chemokines, thus stimulating the migration of macrophages and microglia towards photoreceptor cells [79,80]. Unbalanced homeostasis is the main mechanism that contributes to degenerative retinal disorders [81]. Furthermore, photoreceptors have been shown to present machinery for oxidative phosphorylation in their outer segments, including the electron transport chain and ATP synthetase [67,82,83]. It is believed that this machinery, typical of mitochondria, would be used for the energy needs of visual phototransduction. The increase in the demand for ATP increases the consumption of oxygen, thus increasing ROS production, which in turn causes oxidative stress. The retina is sensitive to oxidative stress and such stress can contribute to diseases such as AMD [83,84]. Polyphenolic compounds can help prevent photoreceptor cell damage caused by ROS, and thus they can have beneficial effects on visual function in retinal degenerative diseases.

The correct function of Rho depends on the correct expression, folding, trafficking, and integration into the lipid bilayer of the cell membrane [85]. Attempts are currently being made to find new ligands that can offset the effects of Rho mutations. Mutations in several genes coding for proteins involved in this visual process can cause retinal diseases, and particularly the retinal degenerative disease RP. Consequently, mechanistic studies of the effects of such mutations on the Rho phenotype are of foremost importance in order to devise effective treatment strategies [81].

### 5.3. Mutations in Rho Associated with Retinal Degenerative Diseases

In the mammalian genome, the ciliary opsin family is made up of different genes, including the Rho gene (RHO), which consists of 5 exons that codify a 348-amino acid protein with a molecular weight of approximately 39 kDa [86].

Pathologies resulting from mutations in the RHO gene can be inherited either as autosomal recessive or autosomal dominant traits. There are two diseases associated with mutations in RHO: congenital stationary night blindness (CSNB) and RP. In the case of CSNB, it is inherited in a dominant way and the term stationary in its name has been questioned because it appears that night blindness could be the first step of a very slowly progressing RP. RP can be inherited both in dominant and recessive ways, although most of the diseases causing mutations are dominant and the recessive phenotype is rare [86].

**Figure 3:**
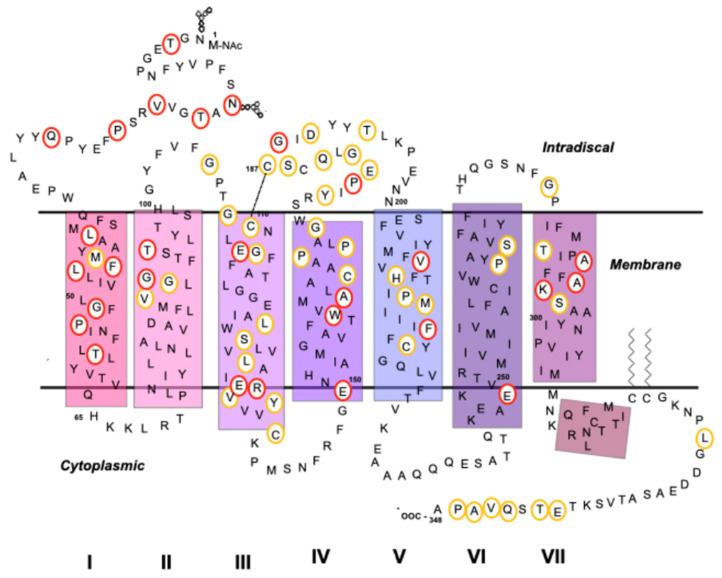
Secondary structure schematic model of Rho, showing amino acids that are sites where mutations associated with RP in patients are found. Sites of RP mutations are circled and those specific positions corresponding to mutations mentioned in the text are circled in red.

Similar phenotypes can result in mutations that affect different regions of the protein, whereas some amino acid changes in the same codon can lead to identical phenotypes but with different severity and RP progression [86]. The first mutation causing RP in the RHO gene was reported at position 23, involving a change from a proline to a histidine (P23H) [87] (a Rho model indicating the site of RP mutations is shown on Figure 3).

Five CSNB-associated missense RHO mutations have been identified: G90D [88,89], T94I [90] E113K [91], A292E [92], and A295V [93]. They are thought to produce a constitutive activation of Rho (except for the E113K mutation) [94]. CSNB mutants have been studied by X-ray crystallography and it has been found that a new salt bridge is formed between the aspartate residue of the G90D mutant and K296 at the retinal binding site. This bridge, and the concomitant breakage of the native salt bridge between E113 and K297, could be the reason for the increased basal activation of this mutant [95]. The constitutive activation of mutants means that they present activity in the absence of the retinal chromophore. Constitutive activity is also referred to Rho activation in the dark, although, in this case, the more precise term of dark activity should be used. The constitutive persistent activation of the phototransduction cascade has been considered a mechanism of cell death in RP [94]. The mutant G90D, which is the cause of CSNB and is a constitutively active mutant, activates the visual cascade without chromophore and in the dark. Interestingly, another mutation at position G90 (G90V) is associated with RP [71,89]. It is an unsolved puzzle why mutations at the same amino acid site cause such distinct clinical phenotypes. The molecular basis of such a striking difference could be related to the stability of quasi-native conformations of opsin (caused by mutations) that would not reflect protein misfolding but rather would affect the conformation equilibrium between active and inactive conformational states [94].

Another mutation that can constitutively activate transducin is the K296E RP mutation [96,97]. Two other mutations associated with CSNB may lead to constitutive transducin activation: A292E and T94I [90,91]. They can do this by disrupting the salt bridge between E113 and K296 that contributes to the stabilization of the protein in its inactive state. Moreover, the T94I CSNB mutant has a hydrophobic side chain that establishes contact with K296 and prolongs the useful lifetime of the active conformation by showing a longer-lived meta II compared to other mutants and the wild-type receptor (WT) [94,98].

The recessive form of RP presents two missense mutations of RHO, namely, E150K [99,100,101] and M253I [102], and two mutations with a premature stop codon, namely, W161ter [103] and E249ter [104]. In contrast, the dominant autosomal inheritance of RP has more than 150 documented mutations, representing 20–30% of all cases, with the P23H mutation being the most studied [105,106]. The biochemical and functional phenotypes of several specific mutations in Rho associated with RP have been previously reported (Table 1).

Mutations found in the *N*-terminal segment of Rho are often associated with mild disease, which develops late and with slowly advancing symptoms. These include P23H, T4K, P23A/L, N15K, T17M, V20G, and Q28H [106,110,111]. The *N*-terminal segment is important because it helps stabilize the retinal-bound conformation of the receptor [86]. Many mutations in the seven transmembrane segments of Rho have been described that can cause different effects on the protein. Many mutations may represent the introduction of a charged amino acid into the membrane domain. These include L40R, L46R, G51R, P53R, and T58R in the first transmembrane helix. The presence of a charged residue may prevent insertion of the domain into the membrane of the endoplasmic reticulum resulting in incorrect folding of the protein [86]. In other cases, RP mutations in the transmembrane helices could result in a loss of side chains necessary for conformational stability and/or functioning or otherwise introduce bulky side chains that may result in steric clashes in densely packed regions of the protein, such as the case of the A164V mutation, which causes an incorrect fold of the protein [112].

An interesting aspect of GPCR functioning is the relevance of receptor–receptor interaction and particularly dimerization and higher-order oligomerization. Rho has the ability to form oligomers [113,114,115], but the functional relevance of such complexes remains to be fully established. In this regard, it has been reported that some mutations have a marked influence on Rho oligomer formation capacity [116]. In the case of the F45, V209, and F220 amino acid positions, found in transmembrane helices 1 and 5, these are the sites of the F45L, V209, and F220C mutations that cannot form dimers or multimers as seen in the case of the WT protein [107,116,117,118].

Two different mutations affect codon 135, where arginine is replaced either by a tryptophan (R135W) or by a leucine (R135L) [119]. These mutations affect an amino acid of the third transmembrane helix at the cytoplasmic membrane boundary. Mutations in codon 135 involve a change in charge and size, a large and basic amino acid is replaced by a non-polar and smaller one in the R135L mutation, and a non-polar, large, and aromatic one in the R135W mutant [119]. E134 and R135 residues are part of the highly conserved D/ERY motif, a site of interaction with the G protein transducin [48,120]. Studies have shown that the R135L and R135W mutations can perform binding in the retina with almost with the same efficiency as in WT cases in reconstituted and purified systems, but they are functionally defective and are not able to efficiently activate transducin [108].

Two other interesting changes occur in the opposite extracellular domain. At the second intradiscal loop, one mutation affects codon 180 and the other affects codon 188, resulting in the P180A and G188R mutations, respectively [109,119]. The substitution of P180A results in the change of a medium-sized hydrophobic residue to a smaller hydrophobic one. The G188R mutation implies the replacement of a small, non-polar amino acid by a large, basic, and positively charged one [121]. These changes can involve both steric and electrostatic effects that can disturb the intradiscal domain packing and the overall conformational stability of the receptor.

A detailed analysis of the structural effects of RP mutations on Rho, as well as the study of genotype-phenotype correlations, is very relevant for elucidating the fine details of the photoreceptor degeneration process. This information is essential to investigate the effects of selected compounds, like polyphenols or specifically flavonoids, on the conformational properties of RP mutant proteins and the subsequent potential clinical benefits of some of these compounds.

## 6. Polyphenols Effects in Retinal Degenerative Diseases

Therapies for retinal degenerative diseases are currently limited, so there is a need to develop new strategies for more effective and safer therapies. Some recent studies have indicated that polyphenols, especially flavonoids, could be viable drug candidates as they may be involved in visual signal transduction and visual pigment regeneration. Flavonoid-rich vegetables and fruits appear to have effects in improving eyesight in eye-related diseases [61,122,123].

We will focus on the effects of flavonoids in three different retinal-related diseases: RP, CSNB and age-related macular degeneration (AMD).

RP includes a heterogeneous set of genetic diseases of the eye that cause vision loss, involving the photoreceptors and the pigment epithelium of the eye [45]. The manifestation of the disease may include loss of night vision and decreased visual field, eventually progressing to blindness. CSNB is a group of heterogeneous genetic disorders of the retina that manifest as non-progressive nyctalopia [124]. Finally, AMD is a complex disease that exhibits several different pathological mechanisms including degeneration of photoreceptors and RPE cells causing visual impairment [125].

Flavonoids such as quercetin and myricetin have been shown to improve the stability of opsin present in rods, increase the binding rate of ligand-free opsin, and facilitate its expression and integration into the membrane in vitro [126]. Because Rho is a critical protein for the structural integrity of the retina, an increase in Rho expression could be a mechanism that contributes to the protective effects of flavonoids that prevent photoreceptor degeneration and retinal deterioration [126]. In spite of the studies presenting beneficial effects of flavonoids, the mechanisms of their protective effects against light-induced retinal damage are not entirely known [123]. Several studies have focused on the effects that flavonoids may exert on the progression of retinal degenerative diseases (Table 2). Some suggest that flavonoids interact directly with Rho, increasing their rates of regeneration, stability, folding, and membrane orientation in vitro [46,127,128].

Flavonoids have been found to stimulate Rho expression, where specifically Rho and cone opsins expression have been improved upon treatment with quercetin and myricetin [127,138,139]. Treatment with quercetin or myricetin in Abca4-/- and Rdh8-/- mice prior to exposure to bright light has been observed to preserve retinal integrity. In this regard, the morphology of the photoreceptors was conserved in those mice that were treated with flavonoids. These results indicate that flavonoids attenuate light-induced receptor damage processes [127].

The antioxidant effect of polyphenols can be invoked as a factor that may delay the progression of AMD. It has been shown that the effects of AMD can be delayed by antioxidants, such as by the antioxidant effects of polyphenols. Polyphenols have also been shown to inhibit ATP synthase at the rod outer segments of the photoreceptor cells. A particular compound, namely, stilbenoid resveratrol, a dietary compound with a wide range of effects on cell function, has also been shown to effectively reduce ROS production, thus protecting against retinal damage [84,140].

Flavonoids can also inhibit inflammatory reactions by suppressing the expression of pro-inflammatory genes and molecules involved in retinal degeneration. It was found that inflammation in the retina induced by bright light in Abca4-/- and Rdh8-/- mice was remarkably reduced with the administration of quercetin and myricetin [127]. Flavonoids can also limit ROS levels by sequestering oxidative radicals. In this regard, RPE cells treated with quercetin could be protected from oxidative stress by inhibiting apoptosis pathways and pro-inflammatory markers [134,141]. Flavonoids enhance the expression of photoreceptor-specific genes by also attenuating the expression of oxidative stress and inflammation-related malignancies and altering the balance between anti-apoptotic and pro-apoptotic genes [129,130,131,135].

Another polyphenol, tannic acid, has been shown to have beneficial effects on health, including the reduced production of UVB-induced interleukin 18 in human keratinocytes, as well as anti-inflammatory properties. Tannic acid has also been reported to inhibit the production of interleukin-6 and to down-regulate the expression of complement factor B in ARPE-19 cells, a factor that is believed to be related to AMD [136].

In ARPE-19 cells, quercetin protects against stress induced by lipid peroxidation [142]. By a reduction in the messenger RNA of proinflammatory interleukins, it also acts by influencing p38, extracellular signal-regulated kinase, mitogen-activated protein kinase, and cAMP response element-binding signaling. These modifications were associated with increased viability of the treated cells. The results indicate that there are several mechanisms by which quercetin can act against oxidative stress in the retina. Similar results were obtained with two other polyphenols: fisetin and luteolin [137]. Quercetin was observed to reduce mitochondrial function protecting against hydrogen peroxide-induced oxidative stress in RPE cells of human donor eyes thus increasing its viability [132]. Other studies have shown that quercetin can improve oxidative stress and its consequences in different regions of the eye [133,143,144,145].

In vivo studies in mice with acquired retinal lesions that were given quercetin did not slow the progression of light-acquired lesions as seen in histological tests; however, they showed a decrease in nitric oxide levels and NADPH/NADP+ ratios and an increase in COX activity and prostaglandin E2 levels, suggesting an antioxidant effect for quercetin [129].

Quercetin has also produced a protective effect against oxidative stress and its consequences on photoreceptor cells resulting from the reaction of ATR with phosphatidylethanolamine producing bis-retinoid photoreactive species [146].

Mechanisms involved in the antioxidant activity of polyphenols include suppression of ROS formation [147,148], thus reducing oxidative damage [149]. The mechanism by which ROS formation is reduced involves phosphorylation of Nrf2 residues resulting in nuclear accumulation [150].

**Figure 4:**
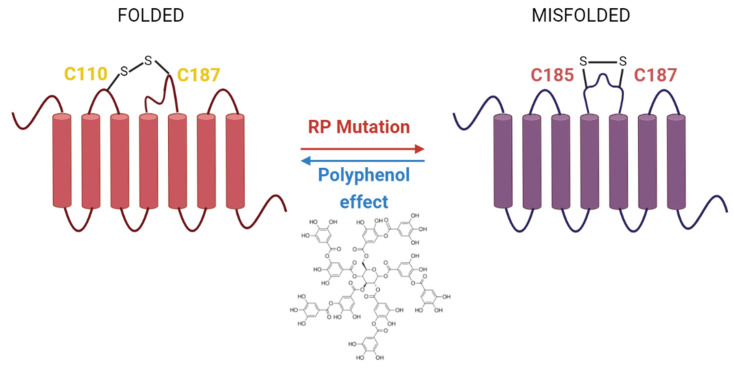
RP mutations and polyphenol effects on Rho conformation. RP mutations can cause misfolded proteins due to abnormal disulfide bonds. In contrast, polyphenols can counteract this effect and fold the protein correctly, thus allowing proper and efficient activity.

Although the implication of flavonoids in vision and vision diseases is still uncertain, some studies with dietary flavonoids like quercetin have suggested potential beneficial effects in some forms of RP [45]. Mutations in Rho are associated with this disease and they can cause protein misfolding that leads to a progressive loss of rod and cone cells, further resulting in vision loss [151,152,153]. These results should be analyzed in the context of research in the RP field, where several strategies based on pharmacological rescue have been proposed for RP treatment. The basic principle of this approach is that chemical or pharmacological chaperones bind to misfolded opsins and are able to stabilize them (Figure 4) [154]. Nowadays, there is an increasing interest in using natural products in order to discover new GPCR ligands that can act as allosteric modulators, and, for example, reduce or compensate the effects generated by RP mutations [45].

The dietary flavonoid quercetin, one of the most studied and widely known for its potential beneficial effects on health [155], has been used in some experiments with the recombinant G90V mutant associated with RP and has shown satisfactory effects when combined with 9-*cis*-retinal (9CR), a retinal analog that is usually employed in vision studies. Over the past years, different investigations have focused on describing the pharmaceutical application of 9-*cis* retinoids to remedy the retinal dysfunction caused by deficient regeneration with 11CR [156,157,158] and have shown that this retinal analog can increase the stability of the RP mutant G90V [70]. As such, a valuable strategy is under development in order to counteract the effects of mutations that cause RP and involves the use of natural products like quercetin alone or in combination with other molecules, like retinal analogs. It is intriguing that in the specific case of amino acid G90, this is the site of the G90V RP mutation and the G90D CSNB mutation, which lead to very different phenotypes. Quercetin was shown to have a positive effect on the binding properties of 9CR containing opsins, especially in the G90V mutant where the rate of regeneration was higher with 9CR than with 11CR. Other properties, such as chemical stability, were also enhanced and some results suggest that there is a synergistic effect as a result of the combination of 9CR and quercetin capable of improving the physicochemical properties of RP mutant proteins. These results suggest that quercetin could act as an allosteric modulator of Rho mutants [45].

In summary, the use of polyphenols, like quercetin, alone or in combination with other small ligands, like retinoids, opens new possibilities for the treatment of retinal degeneration associated with RP. Moreover, the new effect attributed to quercetin may also be applicable to other members of the GPCR superfamily [45]. In spite of these encouraging results, there is clearly a need to further investigate the in vivo potential of such strategies and particularly to increase the number of clinical studies being performed. This is essential to fully determine the exact reach of these newly proposed mechanisms and the potential physiological effects of specific compounds.

## 7. Conclusions

The consumption of food rich in polyphenols has ultimately been related with a range of different health benefits due to their effects on different diseases, and also their impacts on cardiometabolic health and brain functioning. Among all these, effects on different visual diseases have been described. Despite the effects found with the administration of flavonoids, the amounts that reach the eye would be much smaller than those ingested and are expected to be in the nanomolar range. In fact, picomolar concentrations of flavonoids have been detected in mouse eyes and such levels are probably not sufficient to achieve a direct uptake effect. The neuroprotective effect on the retina observed in flavonoids may be associated with its modulating effects on specific cellular pathways related to antioxidant mechanisms, apart from the stabilizing effect of Rho [127].

We have outlined the reported effect of polyphenols on ROS at the outer segment level, in addition to their established role at the inner segment mitochondria. Moreover, a novel aspect of our analysis refers to the potential effect of such compounds by direct interaction with retinal photoreceptor proteins. It is also relevant to note that the conflicting reports on clinical studies conducted to ascertain the potential benefits of these compounds of retinal degenerative diseases prompt further investigation in this field to solve these contradictory findings. The fact that polyphenols are readily metabolized makes their study, and the assignment of a given biological function to a particular chemical species, an arduous and complex task. We would like to stress the notion that, in addition to their known role as antioxidants, polyphenols may have a direct role on receptors, like in the case of Rho and RP. This approach may involve the use of polyphenols, such as quercetin, or combinatorial therapies with other ligands.

The results presented by the different studies reported on the effects of flavonoids support the need of further evaluation of these compounds as the basis for the development of pharmacological treatments against retinal degenerative diseases. There is a clear need for more clinical studies to establish the physiological relevance of the reported effects of polyphenols on visual function. Furthermore, refined characterization of the different Rho mutants and molecular modeling studies are required to better understand the causes of retinal degeneration diseases in humans and to find an efficient approach in order to treat/reduce the symptoms of these diseases.

## Figures and Tables

**Table 1:** Summary of the biochemical phenotypes of selected mutations in Rho associated with retinal degenerative diseases, namely, RP and CSNB.

Mutation	Behavior/Effect	Class/Misfolds	References
G90X	Causes thermal instability and/or abnormal photoproduct formation in inducing a RP phenotype.	VI/No	[45]
T94I	Induces constitutive activation of the opsin in the absence of chromophore and in the dark.	VI/No	[94]
E113K	Associated with the two distinct phenotypes of RP and CSNB in independent members of the same family.	Unclassified	[91]
A292E	Anomalously activates transducin when the chromophore is missing.	Unclassified	[92]
P23H	Destabilizes outer rod segments via the formation of aggregates due to retention in the ER.	II/Yes	[87]
E150	No observed biochemical or cellular defects or not studied in detail.	Unclassified	[101]
W161X	No observed biochemical or cellular defects or not studied in detail.	Unclassified	[103]
G114V	No observed biochemical or cellular defects or not studied in detail.	Unclassified	[107]
Q184P	No observed biochemical or cellular defects or not studied in detail.	Unclassified	[107]
R135X	Affects endocytosis	III/No	[108]
G188R	Forms aggregates due to retention in the ER and cannot be easily constituted with 11CR.	II/Yes	[109]

**Table 2:** Summary of different polyphenols effects on retinal physiology.

Compound	Condition/Cell Lines	Effect	References
Quercetin	Oxidative stress conditions. Assay in vitro in human hepatoma HepG2 cells.	Activates the Nrf2-ARE signaling pathway and exhibits anti-oxidative stress activity alone and together with kaempferol and pterostilbene.	[123]
	Oxidative stress conditions. Assay in vitro in human RPE cells and in *Ccl2/Cx3cr1* double knock-out mice.	Protects RPE cells from oxidative stress via inhibiting pro-inflammatory molecules and the intrinsic apoptosis pathway.	[129]
	VEGF-treated mouse photoreceptor-derived 661W cells.	Inhibits the production of inflammatory proteins in VEGF-stimulated 661W cells.	[130]
	Oxidative stress conditions. ARPE-19 human retinal pigment epithelial cells.	Protects ARPE-19 cells from H_2_O_2_-induced cytotoxicity by activating the Nrf2 pathway, inhibiting ER stress and targeting anti-apoptotic proteins.	[131]
	Oxidative stress conditions. Assay in vitro in human RPE cells.	Protects RPE cells from oxidative damage and cellular senescence in a dose-dependent manner.	[132]
	Oxidative stress conditions. Assay in vitro and in vivo in human RPE cells.	Protects against blue light-induced retinal damage.	[133]
Myricetin	Human MCF-7 breast cancer cells.	Reduces and scavenges intracellular ROS.	[134]
Apigenin	Bright light-exposed BALB/c mice.	Confers retinal protection by inhibiting retinal oxidative stress and retinal inflammatory responses.	[135]
Tannic acid	Assay in vitro in human RPE cells (ARPE-19).	Protects RPE against ultraviolet B radiation via the inhibition of the inflammatory response.	[136]
Fisetin/Luteolin	Assay in vitro in human RPE cells (ARPE-19).	Anti-inflammatory and cytoprotective effects when used as dietary supplements.	[137]

## Abbreviations

11CR	11-*cis*-retinal
9CR	9-*cis*-retinal
AMD	Age-related macular degeneration
ATR	all-*trans*-retinal
cGMP	Cyclic guanosine monophosphate
CSNB	Congenital stationary night blindness
GPCR	G protein-coupled receptor
Gt	G-protein transducin
Meta II	Metarhodopsin II
NCDs	Non-communicable diseases
Rho	Rhodopsin
RHO	Opsin gene
ROS	Reactive oxygen species
RP	Retinitis pigmentosa
RPE	Retinal pigment epithelium
WT	Wild type
